# Supplementary material for: Refractive error and its associated factors among pregnant women attending antenatal care unit at the University of Gondar Comprehensive Specialized Hospital, Northwest Ethiopia
Source: PLoS One. 2021 Feb 12;16(2):e0246174. doi: 10.1371/journal.pone.0246174 (PMC7880455; doi:10.1371/journal.pone.0246174)
Supplement: S1 File — (DOCX) [file pone.0246174.s001.docx]

**ANNEX: written consent form**

Dear respondent my name is _______________________________________, I am here as a data collector. I am going to ask some questions related to socio-demography, health, visual and pregnancy related issues as well as I will examine your BP and the eyes. Your honesty participation in the interview to answer in the questionnaire will provide a valid result on the prevalence of refractive error and factors associated to it. Your genuine response helps us to make appropriate interventions as well; hence we request you to participate honestly. Your volunteer participation in answering the prepared questionnaire and every aspect of the study are completely voluntary. You may stop the interview at any time you want. You may also ask me to clarify questions if you don’t understand it. Finally, all the information that you provide for the study is kept completely confidential. Your responses to our questions are identified only by number, never by name.

Do you agree to participate in this study? If your answer is yes proceed, if no stop here.

Thank for your kindly cooperation.

1. Yes 2. No stop

If you proceed in the study of data collection process,

Signature of the study participant _______________ Date_______________

Name of Data Collector _____________________sign _________Date___________

**English version questionnaire:**

**Code number of the study participant __________**

**I. Socio-demographic characteristics**

| **S/N** | **Variables** | **Response** |
| --- | --- | --- |
| 101 | Age | ____________ years |
| 102 | Religion | 1. Orthodox 2. Muslim 3. Protestant 4. Catholic 5. Others(specify)_________ |
| 103 | Residence | 1. Urban 2. Rural |
| 104 | Educational status | 1. Can’t read and write 2. Grade 8 and lower 3. Grade 9-12 4. > grade 12 |
| 105 | Occupation | 1. Government employee 2. Private employee 3. Merchant 4. House wife 5. Farmer 6. Daily laborers 7. Others specify-------- |
| 106 | Monthly income | _______________________Ethiopian birr |

**II. Medical history – take history and see chart of the pregnant women.**

| 201 | Parity | ______________ |
| --- | --- | --- |
| 202 | Gestational age(in weeks) | ________________weeks |
| 203 | History of DM after pregnancy (GDM) | 1. Yes 2. No |
| 204 | History of HTN after pregnancy (PIH) | 1. Yes 2. No |
| 205 | Family history of spectacle use | 1. Yes 2. No |
| 206 | Using computers or watching TV at least once a day for not less than 2 hours | 1. Yes 2. No |
| 207 | History of medication ( anti-rheumatic, psychiatric & antithrombotic drugs) in the last one month | 1. Yes 2. No |
| 208 | History of contraceptive use before pregnancy | 1. Yes 2. No |
| 209 | How many hours of actual sleep do you get at night? | __________________Hrs. |

**III. Substance use assessment (alcohol intake, khat chewing, cigarette smoking)**

| **301** | Have you ever drunk alcohol in your life time? | 1. Yes 2. No |
| --- | --- | --- |
| **302** | If yes to Q301, have you drink alcohol within the last 30days? | 1. Yes 2.No |
| **303** | Have you ever drunk coffee in your life time? | 1. Yes 2.No |
| **304** | If yes to Q305, have you drink coffee within the last 30days? | 1.Yes 2.No |
| **305** | If yes to Q306, how much cups of coffee you drink per day? | **1**. 1-2 cups **2**. 3-4 cups **3**. ≥ 5 cups |
| **306** | Have you ever smoked cigarette in your life time? | 1.Yes 2. No |
| **307** | If yes to Q308, have you smoked within the last 30days? | 1. Yes 2.No |
| **308** | If yes to Q309, how many cigarettes you smoke daily (in pcs) | ________ |

**IV- PERCEIVED STRESS SCALE**

The questions in this scale ask you about your feelings and thoughts during the last month. In each case, you are asked *to encircle your choice*.

**0 = Never, 1 = Almost Never, 2 = Sometimes, 3 = Fairly Often, 4 = Very Often**

| 401 | In the last month, how often have you been upset because of something that happened unexpectedly? | 0 | 1 | 2 | 3 | 4 |
| --- | --- | --- | --- | --- | --- | --- |
| 402 | In the last month, how often have you felt that you were unable to control the important things in your life? | 0 | 1 | 2 | 3 | 4 |
| 403 | In the last month, how often have you felt nervous and “stressed? | 0 | 1 | 2 | 3 | 4 |
| 404 | In the last month, how often have you felt confident about your ability to handle your personal problems? | 0 | 1 | 2 | 3 | 4 |
| 405 | In the last month, how often have you felt that things were going your way? | 0 | 1 | 2 | 3 | 4 |
| 406 | In the last month, how often have you found that you could not cope with all the things that you had to do? | 0 | 1 | 2 | 3 | 4 |
| 407 | In the last month, how often have you been able to control irritations in your life? | 0 | 1 | 2 | 3 | 4 |
| 408 | In the last month, how often have you felt that you were on top of things? | 0 | 1 | 2 | 3 | 4 |
| 409 | In the last month, how often have you been angered because of things that were outside of your control? | 0 | 1 | 2 | 3 | 4 |
| 410 | In the last month, how often have you felt difficulties were piling up so high that you could not overcome them? | 0 | 1 | 2 | 3 | 4 |

**VI- Physical examination and measurements**

| **S/N** | **Type of measurement** | | **Unit of measurement** |
| --- | --- | --- | --- |
| **501** | Hemoglobin level | | __________g/dl |
| **502** | BP(convenient arm- SBP/DBP) | | SBP_________mmHg |
|  |  |  | DBP________ mmHg |
| **503** | Unaided VA | OD (Oculus Dextrus) _____________ | |
|  |  | OS (Oculus Sinister)______________ | |
| **504** | VA after best correction | OD (Oculus Dextrus) ______________ | |
|  |  | OS (Oculus Sinister)_______________ | |
| **505** | Retinoscopy | OD___________________ | |
|  |  | OS____________________ | |

**የአማርኛ መጠይቅ ቅፅ**

ጎንደር ዩኒቨርሲቲ ጤና ሳይንስ ኮሌጅ

ፊዚዮሎጂ ትምህርት ክፍል

የፈቃደኝነት ማረጋገጫ

እኔ-------------------------------------------------------------------- እባላለሁ፡፡ በጎንደር ዩኒቨርሲቲ ጤና ሳይንስ ኮሌጅ ለሚሰራዉ ጥናት መረጃ ሰብሳቢ ነኝ፡፡ ጥናቱም በጎንደር ዩኒቨርሲቲ ስፔሻላይዝድ ማስተማሪያ ሆሰፒታል በነፍሰ-ጡር እናቶች ላይ የሚደርሰውን የእይታ ስርዓት ጉዳትን የሚያጠና ነዉ፡፡ እርስዎም በጥናቱ ለመካተት ተመርጠዋል፡፡ የሚሰጡትም ምላሽ በሚስጥር የሚያዝ ይሆናል፡፡ ስምዎትም በመጠይቁ ዉስጥ አይጠቀስም፡፡ ፈቃደኛ ካልሆኑ በጥናቱ እንዲሳተፉ አይገደዱም፡፡ በማንኛዉም ጊዜ መጠይቁን ማቋረጥ ይችላሉ፡፡ ነገር ግን በጥናቱ በመሳተፍ የሚሰጡት መረጃ ለነፍሰ-ጡር እናቶች ጤና መሻሻል ወሳኝ ከመሆኑም በላይ የችግሩን ጥንካሬ እና መጠን አዉቆ አፋጣኝ መፍትሄ ለመስጠትና እንዲሁም የተሻለ ፖሊሲ እና ስትራቴጅ በመቅረጽ የሀገራችንን እናቶች ጤና አሁን ካለበት፡የተሻለ ለማድረግ ይረዳል፡፡ በመሆኑም እባክዎትን ትክክለኛ መረጃ መስጠትዎን ይቀጥሉ፡፡ መጠይቁን ለመመለስ የሚወሥደዉ ጊዜ ጥቂት ደቂቃዎችን ብቻ ነዉ፡፡ በማንኛዉም ጊዜ ማብራሪያ ካስፈለገዎ መጠየቅ ይችላሉ ፡፡

በጥናቱ ለመሳተፍ ይስማማሉ ሀ. እስማማለሁ ለ. አልስማማም

መልስዎ እስማማለሁ ከሆነ፡ ይቀጥሉ ፡ አልስማማም ከሆነ ግን እዚህ ላይ ያቁሙ ፡፡

ለተሳትፎ ፈቃደኝነትዎት እጅግ በጣም ከልብ እናመሰግናለን፡፡

የተሳታፊዋ ስምምነት መግለጫ

የዚህ ጥናት ዓላማ፡ ሂደት እንዲሁም ጥቅም ግልጽ ስለሆነልኝ በተጨማሪም የእኔን መብት እና ክብር ሙሉ በሙሉ የጠበቀ ስለሆነና ለሀገርም ጠቃሚ ሆኖ ሰላገኘሁት በጥናቱ ላይ ለመሳተፍ ፈቃደኛ መሆኔን በፊርማየ አረጋግጣለሁ፡፡

የተሳታፊዋ ፊርማ-----------------------------------ቀን--------------------------------------------

የመረጃ ሰብሳቢዉ ስም---------------------------------ፊርማ---------------ቀን------------------

**ክፍል I. ማህበራዊ እና ኢኮኖሚያዊ ሁኔታን የሚመለከቱ ጥያቄዎች፡**

**የጥናቱ ተሳታፊ መለያ ቁጥር____**

| **ተ.ቁ** | **ጥያቄዎች** | **ምላሾች** | | | |
| --- | --- | --- | --- | --- | --- |
| **101** | እድሜ | _________ዓመት | | | |
| **102** | ሃይማኖት | **1.** ኦርቶዶክስ **2**. ሙስሊም **3**.ፕሮቴስታንት **4**. ካቶሊክ **5**. ሌላ ካለ________ | | | |
| **103** | በቋሚነት የሚኖሩበት ቦታ የት ነው? | | | | 1. **1**. ከተማ **2**. ገጠር |
| **104** | የትምህርት ደረጃ | | **1**. ማንበብ እና መፃፍ የማይችል **2**. የመጀመሪያ ደረጃ (< 8) **3**. ሁለተኛ ደረጃ (9-12) 4. ኮሌጅ ወይም ዩኒቨርሲቲ | | |
| **105** | ስራ | | **1**. የመንግስት ሰራተኛ **2**. የግል ሰራተኛ **3**. ነጋዴ **4**. የቤት እመቤት **5**. አርሶ አደር **6**. የቀን ሰራተኛ **7**. ሌላ ካለ___ | | |
| **106** | ወርሃዊ ገቢ | | | _____________ ብር | |

**ክፍል-II. የህክምና ታሪክ ( የታካሚውን የህክምና ቻርት ተመልከት)**

| **201** | እስካሁን ምን ያህል ጊዜ ወልደዋል(የመውለድ ብዛት)? | ______________ |
| --- | --- | --- |
| **202** | የእርግዝናው እድሜ በሳምንት | _____________ሳምነት |
| **203** | በእርግዝና ምክንያት የተከሰተ የስኳር ህመም አለበዎት? | **1.** አዎ **2**. የለም |
| **204** | በእርግዝና ምክንያት የተከሰተ የደም ግፊት አለበዎት? | **1**. አዎ **2**. የለም |
| **205** | ከቤተሰበዎ ውስጥ በእይታ ችግር ምክንያት መነፀር የሚጠቀም አለ? | **1**. አዎ **2**. የለም |
| **206** | በየቀኑ ከሁለት ሰዓት ያላነሰ ኮምፒውተር ይጠቀማሉ ወይም ቴሌቪዥን ያያሉ? | **1**. አዎ **2**. የለም |
| **207** | ባለፈው ወር ውስጥ መድኃኒት (ለቁርጥማት ፣ ለአእምሮ ህመምና ለደም መርጋት) የሚወሰዱ መድሃኒት ወስደዋል? | **1**. አዎ **2**. የለም |
| **208** | ከማርገዘዎ በፊት የወሊድ መቆጣጠሪያ ወስደው ነበር? | 1. አዎ 2. የለም |
| **209** | ምን ያህል ትክክለኛ ሰዓት ማታ ማታ ይተኛሉ ? | ____________ሰዓት |

**ክፍል III. የንጥረ ነገር ሁኔታ ጥያቄዎች (አልኮሆል መጠጥ፣ ጫት መቃም፣ ሲገራ ማጨስ)**

| **301** | በሂዎትዎ አልኮሆል ያለበት መጠጥ ጠጥተው ያውቃሉ? | **1.** አዎ **2**. አላውቅም |
| --- | --- | --- |
| **302** | ለጥየቄ 301 መልሱ አዎ ከሆነ,ባለፉት 30 ቀናት አልኮሆል ተጠቅመወል? | **1**. አዎ **2**. የለም |
| **303** | በሂዎትዎ ቡና ጠጥተው ያውቃሉ? | **1**. አዎ  **2**. የለም |
| **304** | ለጥየቄ 305 መልሱ አዎ ከሆነ,ባለፉት 30 ቀናት ቡና ተጠቅመወል? | **1**. አዎ **2**. የለም |
| **305** | ለጥየቄ 306 መልሱ አዎ ከሆነ,ምን ያህል ስኒ ቡና በቀን ይጠቀማሉ? | **1**. 1-2 ስኒ **2**. 3-4 ስኒ **3**. ≥ 5 ስኒ |
| **306** | በሂዎትዎ ሲጋራ አጭሰው ያውቃሉ? | **1.** አዎ **2.** አላውቅም |
| **307** | ለጥየቄ 308 መልሱ አዎ ከሆነ, ባለፉት 30 ቀናት አጭሰዋል? | **1**. አዎ **2**. የለም |
| **308** | ለጥየቄ 309 መልሱ አዎ ከሆነ,በቀን ምን ያህል ሲጋራ ያጨሳሉ ?(ቁጥር) | _______________ |

**ክፍል IV- የጭንቀት መጠን መለኪያ)**

በዚህ ልኬት ውስጥ ያሉ ጥያቄዎች በመጨረሻው ወር ስለ ስሜቶችዎ እና ሀሳቦችዎ ይጠይቁዎታል። በእያንዳንዱ ሁኔታ ምርጫዎን እንዲያመለክቱ ይጠየቃሉ ፡፡

**0= በጭራሽ፣ 1= መቼም፣ 2= አንዳንድጊዜ፣ 3= ብዙ ጊዜ፣ 4= በጣም ብዙ ጊዜ**

| **401** | ባለፈው ወር ባልተጠበቀ ነገር የተነሳ ምን ያህል ጊዜ ተቆጥተው (ተበሳጭተው) ነበር? | 0 | 1 | 2 | 3 | 4 |
| --- | --- | --- | --- | --- | --- | --- |
| **402** | በአለፈው ወር በሕይወትዎ ውስጥ አስፈላጊ የሆኑትን ነገሮች ለመቆጣጠር እንዳልቻሉ ስንት ጊዜ ይሰማዎታል? | 0 | 1 | 2 | 3 | 4 |
| **403** | ባለፈው ወር ውስጥ ምን ያህል ጊዜ ፍርሃት(በጭንቀት መርበድበድ) እና “ጭንቀት ይሰማዎታል? | 0 | 1 | 2 | 3 | 4 |
| **404** | ባለፈው ወር ውስጥ የግል ችግሮችዎን ለመቆጣጠር በችሎታዎ ስንት ጊዜ ተማምነው ነበር? | 0 | 1 | 2 | 3 | 4 |
| **405** | ባለፈው ወር ውስጥ ነገሮች እርሰዎ በሚፈልጉት መንገድ እየሄዱ እንደሆኑ ስንት ጊዜ ተሰምቶዎት ነበር? | 0 | 1 | 2 | 3 | 4 |
| **406** | ባለፈው ወር ውስጥ ማድረግ ያለብዎትን ነገሮች ሁሉ መቋቋም እንደማይችሉ ምን ያህል ጊዜ ገጥሞዎታል? | 0 | 1 | 2 | 3 | 4 |
| **407** | ባለፈው ወር ውስጥ ብስጭት (ቁጣ) ስንት ጊዜ መቆጣጠር ችለው ነበር? | 0 | 1 | 2 | 3 | 4 |
| **408** | ባለፈው ወር ውስጥ ፣ ነገሮች በሁሉም ነገር ላይ እንደሆኑ የሚሰማዎት ጊዜ ምን ያህል ነበር? | 0 | 1 | 2 | 3 | 4 |
| **409** | ባለፈው ወር ውስጥ ከእርስዎ ቁጥጥር ውጭ በሆኑ ነገሮች የተነሳ ስንት ጊዜ ተቆጥተው ነበር? | 0 | 1 | 2 | 3 | 4 |
| **410** | ባለፈው ወር ውስጥ ችግሮቹ ከባድ ከመሆናቸው የተነሳ መቋቋም(ማሸነፍ) አልቻልኩም ብለው ምን ያህል ጊዜ ተሰምቶዎት ነበር? | 0 | 1 | 2 | 3 | 4 |

**ክፍል V- የአካል ምርመራ እና ሌሎች ልኬቶች - Physical examination and measurements**

| **S/N** | **Type of measurement** | | **Unit of measurement** |
| --- | --- | --- | --- |
| **502** | Hemoglobin level | | __________g/dl |
| **503** | BP(convenient arm- SBP/DBP) | | _____/____mmHg(SBP/ DBP) |
| **504** | Unaided VA | OD (Oculus Dextrus) _________________ | |
|  |  | OS (Oculus Sinister)__________________ | |
| **505** | VA after best correction | OD (Oculus Dextrus) _________________ | |
|  |  | OS (Oculus Sinister)_________________ | |
| **506** | Retinoscoppy | OD_________________ | |
|  |  | OS_________________ | |
