# Supplementary material for: Refractive error and its associated factors among pregnant women attending antenatal care unit at the University of Gondar Comprehensive Specialized Hospital, Northwest Ethiopia
Source: PLoS One. 2021 Feb 12;16(2):e0246174. doi: 10.1371/journal.pone.0246174 (PMC7880455; doi:10.1371/journal.pone.0246174)
Supplement: Attachment — Submitted filename: Reviewers comments_PONE-D-20-30136.docx [file pone.0246174.s002.docx]

**PONE-D-20-30136**

**Overview**: The authors have done a hospital-based cross-sectional study on refractive errors among pregnant women attending antenatal care in Ethiopia to determine prevalence and factors.

**Specific comments**:

*Abstract*:

Page (P)2,Line (L)27: Instead of writing “significantly significant factors” just write significant factors.

Q: Did you consider finding out the rural-urban difference in myopia prevalence among the participants?

Q: Did you consider looking at the time spent outdoors as causative factors in your study?

Q: Considering the myopia prevalence in some countries ranging up to higher 80%, even up to 96.5% in some population group, the refractive error prevalence of 35.66% is not high.

*Main Text*:

Q: Is cataract the commonest cause of blindness and visual impairment in Ethiopia, second being refractive error?

P3, L68: give space after hyperopia.

P4, L70: You need not define disabilities in the bracket. It is understood. Other examples are L46-48, where you have defined the terms in bracket. These are not required. All these redundant or loose writings are grading down your article.

P4, L69-73: Modify this sentence. It is too lengthy, and some link or words are missing before the retinal detachment.

P5, L98: It is not clear what you want to say here.

P5, L99: It is tricky here. Do you mean to say those women more myope than -0.50D, and hyperopia more than +0.50 D were included? You need to be more clear in this sentence.

P6, L126: What is khat?

P7, L144-147: The data collectors were trained so why was it necessary to translate the questionnaire from English to local language and back to English?

P10, L192-194: This sentence does not make any sense or even contrasting your own findings. Please explain.

Q: Your approach seems to have been mainly based on the compensatory mechanism of developing myopia. Currently there is emphasis on the Dopamine theory of myopia development. On this line have you looked if exposure to sunlight or time spent outdoors were related to development of refractive errors?

Q: Rural-urban difference in myopia prevalence in your study and compared with findings in other studies conducted in similar developing countries would be interesting to look at.

P11, L216: What is RE? You have not defined it earlier. You have used refractive errors and RE interchangeably without defining it. If you wish to use abbreviations define it in the first mention and be consistent with it.

P11, L235: Provide space after progesterone.

Q: Are there any limitations in your study that you would like to mention at the end of the Discussion?

*Final appraisal*:

1. Improve writing. You may seek help from professional editing.
2. Rural-urban difference in prevalence of myopia in your study would be interesting.
3. Look for any association of time spent outdoors or sun exposure and refractive error prevalence.
