# Supplementary material for: Refractive error and its associated factors among pregnant women attending antenatal care unit at the University of Gondar Comprehensive Specialized Hospital, Northwest Ethiopia
Source: PLoS One. 2021 Feb 12;16(2):e0246174. doi: 10.1371/journal.pone.0246174 (PMC7880455; doi:10.1371/journal.pone.0246174)
Supplement: Attachment — Submitted filename: Comments to authors.docx [file pone.0246174.s003.docx]

**Comments to authors**

While the study is interesting, the the paper needs improvements throughout all sections. However, the methodology and discussion sections are the ones that need major improvement, as the procedures are not adequately explained and the message is clear. The methodology sections need to be reformulated to emphasize the procedure of clinical data (refraction) as well as visual impairment. The methods/procedure of data collection is described in sufficient detail. Several references are not properly cited and/or are outdated. It needs to be replaced and re-arranged.

**Background**

It would be appropriate to give an global estimate of refractive error(RE), prevalance of RE on pregnancy, imapct, known associations and establish gap.

Lines 44-48: Sentences not necessary . Please delete.

Lines 49-51: Estimates are outdated and reference [5-6] are old. Please use latest estimates (Flaxman SR, 2017, Lancet)

Line 53: Reference 3 is old. Please add recent estimates.

Lines 51-54: The sentence is confusing; as it talks of prevalence in Ethopia and refers to study in Timor-Leste.

Line 55: Please use updated reference and add available reference on prevalence on refractive error in pregancy.

Lines 56-64: This section should go to discussion.

Lines 78-80: The hospital based prevalence may not be adequate to generalise to general population in Ethopia.

**Methods and materials**

Lines 86-87: Why was pegnant women with medical conditions and previous RE excluded? Was women with pre-existing RE excluded from the study. Excluding women with eye problem/RE before will underestimate prevalence of RE in pregant women.

Lines 99-100: Please cite a reference why you chose to consider SE of 0.5 for myopia and hypermetropia.

Lines 106-131: The operational definition may come as a supplementary file. Extensive definition is not necessary. As degrees of RE was not assessed in the study nor discussed in this paper, it need not be mentioned.

132-137: The ‘Data Collection Procedure’ is not adequately described. Please attach the questionnaire and proforma for data collection as as supplement files. Was the questionaire validiated? Since refractive error is the primary outcome measure, please explain in detail how refraction was performed (cycloplegic/non-cycloplegic).

**Results**

Please use decimals uniformly throughout.

Lines 170-175: The paragraph is the repatition of Fig 1 and does provide same results. Please delete either one of it.

**Discussion**

Lines 210-211: If the cut-off pont differs, comparion of RE is not possible. Please compare with those with similar cut-off points.

Lines 212-215: Comparision of RE with normal population of the study site is important. Comparison of pregnant women (only female) from the study site to general population (male/female) from other country is not appropriate.
