# Supplementary material for: Refractive error and its associated factors among pregnant women attending antenatal care unit at the University of Gondar Comprehensive Specialized Hospital, Northwest Ethiopia
Source: PLoS One. 2021 Feb 12;16(2):e0246174. doi: 10.1371/journal.pone.0246174 (PMC7880455; doi:10.1371/journal.pone.0246174)
Supplement: Attachment — Submitted filename: Response to Reviewers.docx [file pone.0246174.s004.docx]

**Point by point response for editor/reviewers comments**

**Manuscript title**: Refractive error and its associated factors among pregnant women attending antenatal care unit at University of Gondar Comprehensive Specialized Hospital, Northwest Ethiopia.

**Manuscript ID**: PONE-D-20-30136

**Dear editor/reviewers**: Thank you for giving us the chance to revise the manuscript. We have addressed all the concerns raised and these modifications are also incorporated in the revised manuscript.

A. **Journal requirements:**

When submitting your revision, we need you to address these additional requirements.

1. Please ensure that your manuscript meets PLOS ONE's style requirements, including those for file naming. The PLOS ONE style templates can be found at <https://journals.plos.org/plosone/s/file?id=wjVg/PLOSOne_formatting_sample_main_body.pdf> and <https://journals.plos.org/plosone/s/file?id=ba62/PLOSOne_formatting_sample_title_authors_affiliations.pdf>

**Author’s response**: We have prepared the manuscript based on PLOS ONE's style requirements.

2. In your Methods section, please provide additional information about the participant recruitment method and the demographic details of your participants. Please ensure you have provided sufficient details to replicate the analyses such as:

- A description of any inclusion/exclusion criteria that were applied to participant recruitment,
- A table of relevant demographic details,
- A statement as to whether your sample can be considered representative of a larger population,
- A description of how participants were recruited, and
- Descriptions of where participants were recruited and where the research took place.

**Author’s response**: All information how and where our participants were recruited, demographic details of the participants and the inclusion/exclusion criteria were included in the revised manuscript. Our sample was sufficient to draw conclusions for our source population. We have calculated the sample size based on single population proportion formula by using a proportion (P) of 0.5 to get the largest possible minimum adequate sample size since there were no previous studies in Ethiopia. We have also used random sampling technique to recruit each study participant. Therefore, our sample was representative of the target population (pregnant women who visited ANC service of the University of Gondar Compressive Specialized Hospital).

3. PLOS requires an ORCID ID for the corresponding author in Editorial Manager on papers submitted after December 6th, 2016. Please ensure that you have an ORCID ID and that it is validated in Editorial Manager. To do this, go to ‘Update my Information’ (in the upper left-hand corner of the main menu), and click on the Fetch/Validate link next to the ORCID field. This will take you to the ORCID site and allow you to create a new ID or authenticate a pre-existing ID in Editorial Manager. Please see the following video for instructions on linking an ORCID ID to your Editorial Manager account: <https://www.youtube.com/watch?v=_xcclfuvtxQ>

**Author’s response**: ORCID ID for the corresponding author was created which is 0000-0003-4186-4991, and the link to public record is <https://orcid.org/0000-0003-4186-4991>.

4. Your ethics statement should only appear in the Methods section of your manuscript. If your ethics statement is written in any section besides the Methods, please move it to the Methods section and delete it from any other section. Please ensure that your ethics statement is included in your manuscript, as the ethics statement entered into the online submission form will not be published alongside your manuscript.

**Author’s response**: The ethics statement was moved to the methods section.

B. **Response to reviewers comments**

**For reviewer # 1**

1. Is the manuscript presented in an intelligible fashion and written in Standard English? You may seek help from professional editing

**Author’s response**: Thank you. We have made extensive English editing throughout the document.

2. Page (P) 2, Line (L) 27: Instead of writing “significantly significant factors” just write significant factors.

**Author’s response**: Thank you. We have deleted the word “significantly” as suggested (page (P) 1, Line (L)-28).

3. Did you consider finding out the rural-urban difference in myopia prevalence among the participants? Rural-urban difference in myopia prevalence in your study and compared with findings in other studies conducted in similar developing countries would be interesting to look at.

**Author’s response**: Thank you for raising this important point. We have checked the association between residence and our outcome variable (RE) in bivariable and multivariable logistic regression analysis. However, the adjusted odds ratio of the variable residence was 0.76 with 95% CI of 0.21-2.77 in our final analysis model. Since the 95% CI includes 1, the association of residence to RE is non-significant (p>0.05) and showed that there is no as such significant difference between urban and rural residents in regarding to RE. Hence, considering residence as stratifying variable will not add new information.

4. Did you consider looking at the time spent outdoors as causative factors in your study? Your approach seems to have been mainly based on the compensatory mechanism of developing myopia. Currently there is emphasis on the Dopamine theory of myopia development. On this line have you looked if exposure to sunlight or time spent outdoors were related to development of refractive errors?

**Author’s response**: Thank you. We didn’t include the time spent outdoors as causative factors directly in our study. However, we have tried to assess its impact based on the occupation of participants, which is highly related to their time spent outdoors. Unfortunately, the variable occupation was not statistically significant (P>0.05) (see table 3) in our final analysis model. We have also put it as limitation of the study (P13, L315-316).

5. Considering the myopia prevalence in some countries ranging up to higher 80%, even up to 96.5% in some population group, the refractive error prevalence of 35.66% is not high.

**Author’s response**: Thank you. Even though the overall prevalence of refractive error in our study is lower than the previous studies mentioned, still it is higher than what is intended to be in 2020 (2.9%). Besides, the prevalence of myopia in our study was 64.34% (P9, L202) which is fairly similar with the numbers you mentioned. Therefore, the prevalence of refractive error and myopia in our study were high.

6. Is cataract the commonest cause of blindness and visual impairment in Ethiopia, second being refractive error?

**Author’s response**: Thank you very much. Cataract is the leading cause of both blindness (49.9%) and visual impairment (42.3%). Refractive error is the second cause for visual impairment (33.4%) but the third cause of blindness (7.8%) in Ethiopia. We have also stated this sentence in the revised manuscript (P3, L50-52).

7. P3, L68: give space after hyperopia.

**Author’s response**: Thank you. We have modified it based on your suggestion (P3, L59).

8. P4, L70: You need not define disabilities in the bracket. It is understood. Other examples are L46-48, where you have defined the terms in bracket. These are not required. All these redundant or loose writings are grading down your article.

**Author’s response**: Thank you. We have removed all of the terms in bracket in the revised manuscript.

9. P4, L69-73: Modify this sentence. It is too lengthy, and some link or words are missing before the retinal detachment.

**Author’s response**: Thank you very much. We have modified the sentence in the revised manuscript as suggested (P3, L61-65).

10. P5, L98: It is not clear what you want to say here.

**Author’s response**: Thank you. It is to mean that our outcome variable was refractive error in which the participants were considered to have refractive error, if they were either myopic (the spherical equivalent ≤ –0.50 dioptres) or hyperopic (the spherical equivalent ≥ +0.50D) or both problems in either or both eyes. If the spherical equivalent (SE) is in between -0.50D and +0.50D in either or both eyes, they are considered as normal. We have also included this modification in the revised manuscript (P6, L106-110).

11. P5, L99: It is tricky here. Do you mean to say those women more myope than -0.50D, and hyperopia more than +0.50 D were included? You need to be clearer in this sentence.

**Author’s response**: Thank you. We have modified the statement to make it more clearly to readers (P6, L106-110).

12. P6, L126: What is khat?

**Author’s response**: Thank you. Khat (Catha edulis) is a flowering plant native to Ethiopia which contains the alkaloid cathinone, a neuro-stimulant substance. For clarification, we stated as “Catha edulis” as an alternative name to Khat in the revised manuscript (P6, L113).

13. P7, L144-147: The data collectors were trained so why was it necessary to translate the questionnaire from English to local language and back to English?

**Author’s response**: Thank you. This was done to check the meaning consistency of the questioner. The mother tongue for data collectors and the participants is Amharic language. Therefore, to create common understanding and to avoid any possible misinterpretation during the interview, we prefer to translate it into Amharic language.

14. P10, L192-194: This sentence does not make any sense or even contrasting your own findings. Please explain.

**Author’s response**: Thank you. it is to mean that study participants with grade 9-12 education level had 0.1 times reduced odds of RE compared to the reference group. As we stated in the manuscript, the above interpretation can also be expressed as study participants with 9-12 education level had 90% (AOR=0.10, 95%CI: 0.02-0.54) reduced odds of refractive error than those who cannot read and write (P13, 18-19, L220-222, 297-304). Therefore, our interpretation is in line with our finding.

15. P11, L216: What is RE? You have not defined it earlier. You have used refractive errors and RE interchangeably without defining it. If you wish to use abbreviations define it in the first mention and be consistent with it.

**Author’s response**: Thank you very much. We have used RE just to say refractive error and now we have corrected it as you suggested in the revised document.

16. P11, L235: Provide space after progesterone.

**Author’s response**: Thank you. We have corrected based on your suggestion (P17, L272).

17. Are there any limitations in your study that you would like to mention at the end of the Discussion?

**Author’s response**: Thank you. As we stated in the last paragraph of discussion section, the present study has the following limitations (P19, L305-311). First, the study was cross-sectional study and hence we are unable to show cause-effect relationship between independent variables and refractive error. Second, we didn’t measurement the axial length of the eyes of study participants that may affect our outcome variable due to resource scarcity. Third, we didn’t apply cycloplegic refraction test to regulate accommodation ability of the lens because of its least feasibility in our institution even though its effect is very little. Lastly, we did not collect data regarding sunlight exposure level and time spent in the outdoor which might have an effect on the outcome variable.

**For Reviewer #2**

1. Is the manuscript technically sound, and do the data support the conclusions?

**Author’s response**: Thank you. We have tried to make the manuscript more technically sound and our data supports our conclusion in the revised manuscript.

2. Is the manuscript presented in an intelligible fashion and written in Standard English?

**Author’s response**: Thank you. We have made extensive English editing throughout the document.

3. The paper needs improvements throughout all sections. However, the methodology and discussion sections are the ones that need major improvement, as the procedures are not adequately explained and the message is clear. The methodology sections need to be reformulated to emphasize the procedure of clinical data (refraction) as well as visual impairment. The methods/procedure of data collection is described in sufficient detail. Several references are not properly cited and/or are outdated. It needs to be replaced and re-arranged.

**Author’s response**: Thank you very much. As we can see in the track changes of the document, we tried to address all the comments and included these corrections in the revised manuscript.

4. It would be appropriate to give an global estimate of refractive error(RE), prevalance of RE on pregnancy, imapct, known associations and establish gap.

**Author’s response**: Thank you. We have reorganized it based on your suggestion (P3-4, L45-77).

5. Lines 44-48: Sentences not necessary . Please delete.

**Author’s response**: Thank you. We have deleted the sentence in the revised manuscript.

6. Lines 49-51: Estimates are outdated and reference [5-6] are old. Please use latest estimates (Flaxman SR, 2017, Lancet)

**Author’s response**: Thank you. We have corrected the reference as suggested (P3, L46).

7. Line 53: Reference 3 is old. Please add recent estimates.

**Author’s response**: Thank you. We had modified the references in the revised manuscript (P3, L49)

8. Lines 51-54: The sentence is confusing; as it talks of prevalence in Ethopia and refers to study in Timor-Leste.

**Author’s response**: Thank you very much. Sorry for the mistake we made. The relevant reference was cited and we have also rewrite the sentence to make it clear in revised manuscript (P3, L50-52).

9. Line 55: Please use updated reference and add available reference on prevalence on refractive error in pregancy.

**Author’s response:** Thank you. We had made corrections regarding your suggestion in the revised manuscript (P3, L53-60).

10. Lines 56-64: This section should go to discussion.

**Author’s response**: Thank you. Modification was mad based on your suggestion and the paragraph was incorporated in the discussion section.

11. Lines 78-80: The hospital based prevalence may not be adequate to generalise to general population in Ethopia.

**Author’s response**: Thank you. We accepted your comment and the word “country” was replaced by “study setting” (P4, L77).

12. Lines 86-87: Why was pegnant women with medical conditions and previous RE excluded? Was women with pre-existing RE excluded from the study. Excluding women with eye problem/RE before will underestimate prevalence of RE in pregant women.

**Author’s response**: Thank you. We excluded the pregnant women with the above medical conditions before pregnancy (diabetes and hypertension before being pregnant) because these conditions are known to increases the chance of having refractive error. We also excluded pregnant women who had RE before pregnancy. This is because our aim was determining the prevalence of RE during pregnancy. Actually the numbers of pregnant women who had RE before pregnancy and excluded in this study were only 3. Therefore, excluding these women may not significantly underestimate the outcome variable.

13. Lines 99-100: Please cite a reference why you chose to consider SE of 0.5 for myopia and hypermetropia.

**Author’s response**: Thank you. Relevant references were cited as suggested (P6, L110).

14. Lines 106-131: The operational definition may come as a supplementary file. Extensive definition is not necessary. As degrees of RE was not assessed in the study nor discussed in this paper, it need not be mentioned.

**Author’s response**: Thank you very much. We have tried to minimize and merge the operational definitions as suggested in the revised manuscript (P7, L116-135).

15. 132-137: The ‘Data Collection Procedure’ is not adequately described. Please attach the questionnaire and proforma for data collection as as supplement files. Was the questionaire validiated? Since refractive error is the primary outcome measure, please explain in detail how refraction was performed (cycloplegic/non-cycloplegic).

**Author’s response**: Thank you. We used a validated questioner which was prepared from different literatures after extensive searching. We performed a non-cycloplegic refraction because of its least feasibility in our setup. Cycloplegic refraction was necessarily indicated for patients suspected for pseudomyopia, accommodative esotropia and for those aged less than 20 years. In our study, most of the participants were aged more than 22 years and hence the effect of accommodation for outcome variable is less significant. There was also no indications to perform cycloplegic test in our study participants. We have included all the concerns raised in the revised manuscript (P7-8, L136-148).

16. Please use decimals uniformly throughout the result section.

**Author’s response**: Thank you. As you can see in the track change document, we have made the decimals uniform throughout the revised manuscript.

17. Lines 170-175: The paragraph is the repatition of Fig 1 and does provide same results. Please delete either one of it.

**Author’s response**: Thank you. We have deleted the figure as suggested.

18. Lines 210-211: If the cut-off pont differs, comparion of RE is not possible. Please compare with those with similar cut-off points.

**Author’s response**: Thank you. In our discussion section, we had modified the cut-off point as a means of discrepancy in the revised manuscript (P16, L245-248).

19. Lines 212-215: Comparision of RE with normal population of the study site is important. Comparison of pregnant women (only female) from the study site to general population (male/female) from other country is not appropriate.

**Author’s response**: Thank you. Since there are limited previous studies on the prevalence of refractive error among pregnant women, we are forced to discuss our finding with the finding non pregnant women and the general population. In the absence of sufficient studies in the area, we hope, comparing our finding with the above population will be important to appreciate the difference and take intervention accordingly. If you are not convinced with our argument, it is possible to delete that part.
