# Supplementary material for: Refractive error and its associated factors among pregnant women attending antenatal care unit at the University of Gondar Comprehensive Specialized Hospital, Northwest Ethiopia
Source: PLoS One. 2021 Feb 12;16(2):e0246174. doi: 10.1371/journal.pone.0246174 (PMC7880455; doi:10.1371/journal.pone.0246174)
Supplement: Attachment — Submitted filename: Comments.docx [file pone.0246174.s005.docx]

Comments

The manuscript is significantly improved; however, several areas can still be improved.

Abstract:

Lines 16-17: ‘such as retinal detachment, difficulty of performing daily living activities and economic crisis’ this can be removed or rephrased.

Line 18: remove the word ‘Therefore’.

Line 30: ‘27 years with inter-quartile range of 24 to 31 years’ can be written as ’27 (IQR 24-31) years.

**Manuscript**

1. There are still a lot of grammatical errors. Please correct.
2. Use the abbreviation of ‘University of Gondar Comprehensive Specialized Hospital’ throughout the manuscript after its first mention in line 75-76.
3. Line 46-48: Statement not appropriate to this study. Can delete.
4. Line 141-142: Delete the line as its explained in lines 142-145.
5. Line 151: Pre-test = Pilot?
6. Line 175: Can you replace the word ‘linked’ with more appropriate word.
7. Line 179-180: Rephrase the sentences and correct the grammar.
8. Lines 240-242: Delete the lines as its already stated in introduction.
9. In the discussion, use odds ratios to describe significance rather than %.
10. Conclusion could be the past paragraph of the discussion.

**Reference:**

Reference 4: Please correct citation style.
