# Supplementary material for: Refractive error and its associated factors among pregnant women attending antenatal care unit at the University of Gondar Comprehensive Specialized Hospital, Northwest Ethiopia
Source: PLoS One. 2021 Feb 12;16(2):e0246174. doi: 10.1371/journal.pone.0246174 (PMC7880455; doi:10.1371/journal.pone.0246174)
Supplement: Attachment — Submitted filename: Response to Reviewers.docx [file pone.0246174.s006.docx]

**Point by point response for reviewers’ comments	Date: Dec 21, 2020**

**Manuscript title**: Refractive error and its associated factors among pregnant women attending antenatal care unit at University of Gondar Comprehensive Specialized Hospital, Northwest Ethiopia.

**Manuscript ID**: PONE-D-20-30136R1

**Dear editor/reviewers**: Thank you for giving us the chance to revise the manuscript. We have addressed all the concerns raised and these modifications are also incorporated in the revised manuscript.

**Response to reviewers’ comments**

**For reviewer # 1**

1. Just a small point: in the revised manuscript under Limitations of the study section, the second limitation you have mentioned is that you did not measure the axial length which might have affected the outcome variables. I do not think this would affect at all because you are not defining the refractive error by axial length in your study.

**Author’s response**: Thank you. We have agreed with your comment and removed it from the revised manuscript.

**For reviewer # 2**

1. Is the manuscript presented in an intelligible fashion and written in standard English?

**Author’s response**: Thank you. We have made an extensive English editing throughout the revised document to improve the quality of our paper.

2. Lines 16-17: ‘such as retinal detachment, difficulty of performing daily living activities and economic crisis’ this can be removed or rephrased.

**Author’s response**: Thank you. We have removed the lists in the revised manuscript.

3. Line 18: remove the word ‘Therefore’.

**Author’s response**: Thank you. We have removed it as suggested.

4. Line 30: ‘27 years with inter-quartile range of 24 to 31 years’ can be written as ’27 (IQR 24-31) years.

**Author’s response**: Thank you. We have rewritten it based on your suggestion.

5. There are still a lot of grammatical errors. Please correct.

**Author’s response**: Thank you. We have tried to correct grammatical errors throughout the revised manuscript.

6. Use the abbreviation of ‘University of Gondar Comprehensive Specialized Hospital’ throughout the manuscript after its first mention in line 75-76.

**Author’s response**: Thank you. We have corrected as suggested in the revised manuscript.

7. Line 46-48: Statement not appropriate to this study. Can delete.

**Author’s response**: Thank you. We have removed the unnecessary statement as suggested in the revised manuscript.

8. Line 141-142: Delete the line as its explained in lines 142-145.

**Author’s response**: Thank you. We have deleted it in the revised manuscript as suggested.

9. Line 151: Pre-test = Pilot?

**Author’s response**: Thank you. We had conducted a pre-test not a pilot study, to assess the reliability and validity of the survey instruments/questioners prior to the actual data collection.

10. Line 175: Can you replace the word ‘linked’ with more appropriate word.

**Author’s response**: Thank you. We have replaced the word ‘linked’ with “referred” in the revised manuscript (Line 170).

11. Line 179-180: Rephrase the sentences and correct the grammar.

**Author’s response**: Thank you. We have rephrased and corrected the grammar as suggested.

12. Lines 240-242: Delete the lines as its already stated in introduction.

**Author’s response**: Thank you. We have removed the statement in the revised manuscript as suggested.

13. In the discussion, use odds ratios to describe significance rather than %.

**Author’s response**: Thank you. We have modified our description of association in the discussion section of the revised manuscript.

14. Conclusion could be the past paragraph of the discussion.

**Author’s response**: Thank you. We have placed the limitations of the study at the last paragraph of the discussion rather than conclusion. This is because the guideline of PLOS ONE recommends the conclusion to be placed separately just next to the discussion section.

15. Reference 4: Please correct citation style.

**Author’s response**: Thank you. The citation style we used for all references was Vancouver, including reference 4. Reference 4 is a guideline with no volume and issue numbers. Since this reference did not add extra information beyond references 2 and 3, we have removed it in the revised manuscript
