# Supplementary material for: Refractive error and its associated factors among pregnant women attending antenatal care unit at the University of Gondar Comprehensive Specialized Hospital, Northwest Ethiopia
Source: PLoS One. 2021 Feb 12;16(2):e0246174. doi: 10.1371/journal.pone.0246174 (PMC7880455; doi:10.1371/journal.pone.0246174)
Supplement: Attachment — Submitted filename: Response to Reviewers.docx [file pone.0246174.s007.docx]

**Response for reviewers’ comment	Date: Jan 12, 2021**

**Manuscript title**: Refractive error and its associated factors among pregnant women attending antenatal care unit at the University of Gondar Comprehensive Specialized Hospital, Northwest Ethiopia.

**Manuscript ID**: PONE-D-20-30136R2

**Dear editor/reviewers**: Thank you for giving us the chance to revise the manuscript. We have addressed all the concerns raised and these modifications are also incorporated in the revised manuscript.

**Response to reviewers’ comment**

**For reviewer # 2**

1. Is the manuscript presented in an intelligible fashion and written in standard English?

 All comments have been addressed. The manuscript can be accepted for publication.
However, minor grammatical errors can still be corrected.

**Author’s response**: Thank you. We have made an English editing throughout the document to improve grammatical errors as suggested.
